# Transthyretin Cardiac Amyloidosis Presenting as Bradycardia, Renal Failure, Atrioventricular-Nodal Blockade, Shock, and Hyperkalemia (BRASH) Syndrome: A Case Report

**DOI:** 10.7759/cureus.44532

**Published:** 2023-09-01

**Authors:** Nazima Khatun, Bernard Brown, Jonathan Francois, Adam S Budzikowski, Louis Salciccioli, Sabu John

**Affiliations:** 1 Department of Internal Medicine, State University of New York Downstate Health Sciences University, Brooklyn, USA; 2 Department of Cardiology, State University of New York Downstate Medical Center, Brooklyn, USA; 3 Department of Cardiology, State University of New York Downstate Health Sciences University, Brooklyn, USA; 4 Department of Cardiology, Kings County Hospital Center, Brooklyn, USA

**Keywords:** shock, heart failure, bradycardia renal failure av nodal blocker, transthyretin cardiac amyloidosis, brash syndrome

## Abstract

BRASH syndrome involves the chain of events resulting from the collective effects of *B*radycardia, *R*enal failure, *A*trioventricular (AV)-nodal blockade, *S*hock, and *H*yperkalemia. BRASH syndrome can rapidly progress to cardiac arrest. Early recognition is crucial. We present a case of transthyretin cardiac amyloidosis (ATTR-CA) in an elderly woman who presented with BRASH syndrome shortly after an AV-nodal blocker was prescribed for atrial fibrillation.

## Introduction

BRASH (*B*radycardia, *R*enal failure, *A*trioventricular (AV)-nodal blockade, *S*hock, and *H*yperkalemia) syndrome is becoming a common cause of significant bradycardia, especially in elderly patients with multiple comorbidities requiring polypharmacy. Although AV-nodal blockade and hyperkalemia can lead to bradycardia by themselves, they can collectively lead to critical bradycardia and decrease cardiac output, compromising renal perfusion and worsening hyperkalemia. This cascade of events ultimately causes multiorgan dysfunction and shock [1,2]. Other potential triggers may include hypovolemia or any event promoting hyperkalemia or renal injury [2,3].

Cardiac amyloidosis is characterized by the deposition of amyloid fibrils in the heart, and those fibrils are usually composed of amyloid light chains or amyloid transthyretin [4,5]. Transthyretin cardiac amyloidosis is classified into two forms, wild type (ATTRwt-CA) and variant type (ATTRv-CA), depending on the presence of genetic mutations in transthyretin [4]. Wild-type ATTR‐CA is caused by age‐related misfolding of transthyretin. Hereditary or variant ATTR‐CA is an autosomal‐dominant disease in which gene mutations lead to changes in the protein transthyretin [6]. The incidence of cardiac amyloidosis has been increasing in the aging population and commonly presents with heart failure, atrial fibrillation/flutter, and heart block [3,5,6]. Early diagnosis is important, as cardiac amyloidosis is progressive and life-threatening if left untreated, and most goal-directed medical therapy for heart failure may cause serious side effects [5,6].

In this case, BRASH syndrome developed soon after starting the AV-nodal blocking agent metoprolol for atrial fibrillation due to various underlying factors enhancing the effect of metoprolol, such as advanced age, acute kidney injury, and concurrent use of sacubitril/valsartan, in the setting of previously undiagnosed ATTR-CA.

This article was previously presented as a conference abstract at the American College of Cardiology Together With World Congress of Cardiology (ACC Together With WCC or ACC.23/WCC) annual scientific meeting in New Orleans, Louisiana, on March 4, 2023.

## Case presentation

A 76-year-old African-American woman presented to the emergency department with a history of worsening generalized edema, fatigue, and shortness of breath. Upon arrival, the patient was afebrile, heart rate of 39 beats per minute, blood pressure of 83/38 mmHg with mean arterial pressure (MAP) of 53 mmHg, oxygen saturation of 99% on room air, and respiratory rate of 20 breaths/min. The patient appeared lethargic but alert and oriented. Physical examination revealed jugular venous distension, bibasilar lung crackles, generalized pitting edema, and cold extremities with 1+ peripheral pulses bilaterally.

The patient had a past medical history of hypertension, hyperlipidemia, chronic kidney disease stage 3, heart failure with an ejection fraction of 27%, and atrial fibrillation. Atrial fibrillation was diagnosed two months prior to this hospitalization when metoprolol tartrate 25 mg twice daily was started. Other home medications included apixaban 5 mg twice daily, dapagliflozin 10 mg daily, sacubitril/valsartan 24-26 mg twice daily, furosemide 80 mg once daily, and aspirin 81 mg once daily. Family history included hypertension and diabetes in the mother and hypertension in the father. Any family history of heart disease was denied.

Initial laboratory tests were significant for elevated troponin, brain natriuretic peptide, serum creatinine (patient’s baseline creatinine was 1.7-1.9 ng/mL), blood urea nitrogen, serum potassium level, and lactate without acidosis. Detailed laboratory results are listed in Table 1.

**Table 1:** Initial laboratory results T4, thyroxine; pCO2, partial pressure of carbon dioxide; pO2, partial pressure of oxygen; HCO3, bicarbonate; FLC, free light chains

Laboratory tests	Results	Reference range
Sodium	133	136 - 145 mmol/L
Potassium	6.4	3.5 - 4.8 mmol/L
Magnesium	3	1.6 - 2.6 mg/dL
Phosphorus	4.1	2.3 - 4.7 mg/dL
Chloride	104	98 - 107 mmol/L
Carbon dioxide	25	22 - 29 mmol/L
Glucose	105	77 - 100 mg/dL
Blood urea nitrogen	68	7 - 20 mg/dL
Creatinine	3.4	0.6 - 1.1 mg/dL
Glomerular filtration rate	13	>= 60 ml/min/1.73 m2
Total protein	6.9	6.7 - 8.6 g/dL
Total bilirubin	1.4	0.2 - 1.2 mg/dL
Aspartate transaminase	26	5 - 34 U/L
Alanine transaminase	8	0 - 37 U/L
Alkaline phosphatase	61	40 - 150 U/L
Calcium	9.7	8.4 - 10.4 mg/dL
Troponin T	0.15	<0.04 ng/mL
B-type natriuretic peptide	2272	<100 pg/mL
Thyroid-stimulating hormone	4.90	0.270 - 4.200 uIU/mL
T4 Free	1.01	0.71 - 1.85 ng/dL
Creatinine kinase	71	29 - 168 U/L
Prothrombin time	29	10.8 - 13.7 sec
International normalized ratio	2.3	
Activated partial thromboplastin time	36	25.4 - 38.6 sec
White blood cell count	4.96	3.80 - 10.80 K/uL
Hemoglobin	10.1	12 - 16 g/dL
Hematocrit	33	34 - 45%
Platelet count	199	150 - 400 K/uL
Venous blood pH	7.32	7.31 - 7.41
Venous blood pCO2	46.5	30 - 50 mmHg
Venous blood pO2	24.7	40 - 52 mmHg
Venous HCO3	23.3	23 - 28 mmol/L
Lactate	2.2	0.5 - 1.6 mmol/L
Serum k-FLC	126.9	3.3 - 19.4 mg/L
Serum λ-FLC	97.2	5.7 - 26.3 mg/L
Serum FLC k/ λ ratio	1.31	0.26 - 1.65
Urine k-FLC	18.31	<= 32.90 mg/L
Urine λ-FLC	3.95	<= 3.79 mg/L
Urine FLC k/ λ ratio	4.64	<= 8.69
Serum protein electrophoresis	Negative	
Urine protein electrophoresis	Negative	

**Figure 1:**
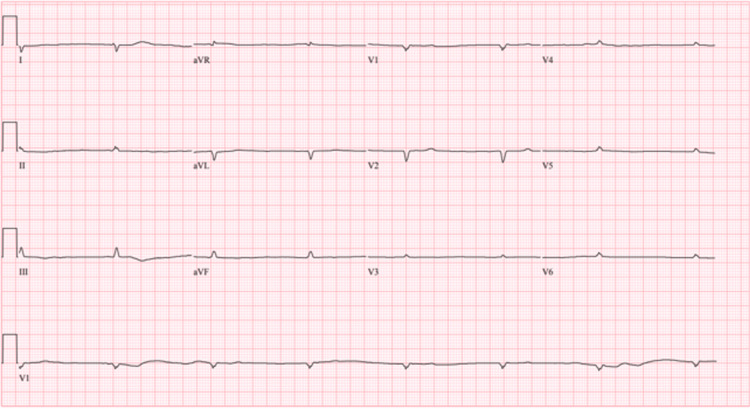
Initial electrocardiogram (ECG) Showing low-voltage QRS complexes with junctional escape rhythm at a rate of 43 beats per minute.

An initial electrocardiogram (ECG) exhibited low voltage QRS, no discernible P wave, ventricular escape rhythm with a ventricular rate of 34 bpm, and a QRS duration of 120 milliseconds (ms) (Figure 1).

**Figure 2:**
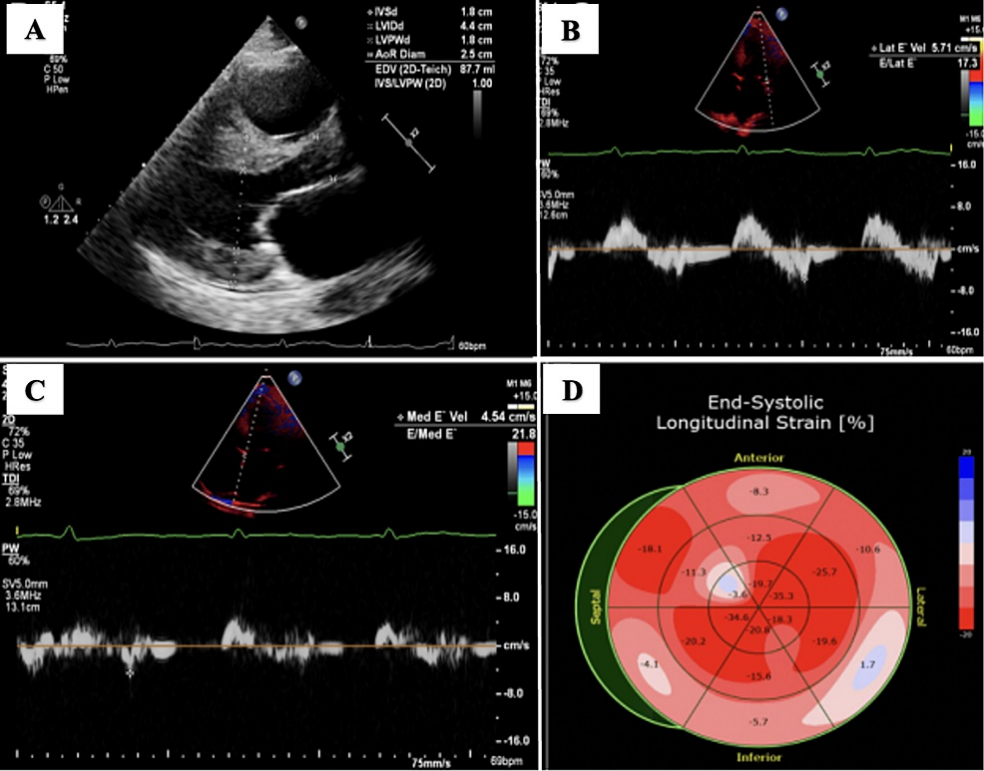
Transthoracic echocardiography (TTE) images A: Parasternal long-axis view showing severe concentric left ventricular hypertrophy. B: Tissue Doppler showing reduced lateral E’ velocity of 5.71 cm/s and elevated E/lateral E’ of 17.3. C: Tissue Doppler showing reduced medial E’ velocity of 4.54 cm/s and elevated E/medial E’ of 21.8. D: Global longitudinal strain score of -16% with apical sparing.

Chest X-ray showed cardiomegaly and bilateral pulmonary vascular congestion. Transthoracic echocardiography (TTE) was significant for severe left ventricular concentric hypertrophy with an ejection fraction (EF) of <30% (Figure 2: A, B, and C). Strain analysis on TTE showed a global longitudinal strain score of -16% with apical sparing (Figure 2: D). 

**Figure 3:**
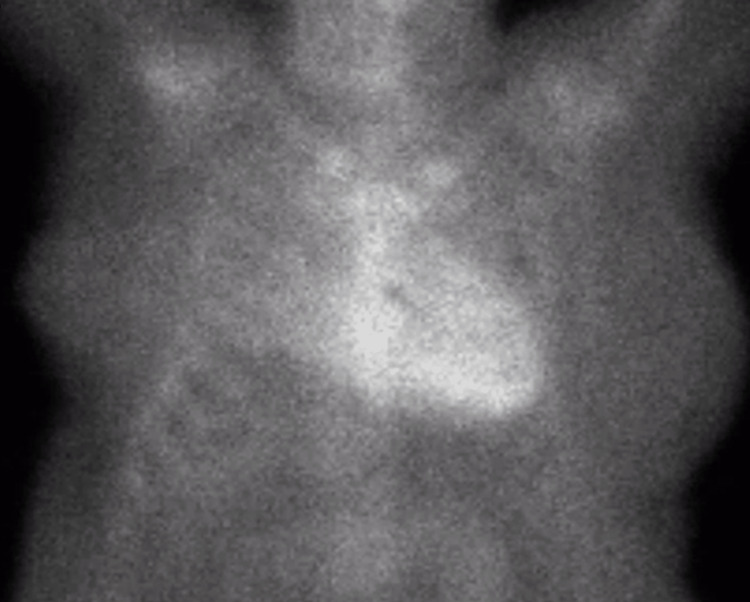
99m-Technetium-pyrophosphate scintigraphy Showing a heart-to-contralateral lung ratio of 1.76 at three hours

Serum and urine immunofixation tests were unremarkable, and serum-free light-chain assays showed a normal kappa-to-lambda ratio of 1.17 (reference range: 0.26-1.65). Technetium-99m-pyrophosphate planar scintigraphy revealed findings consistent with cardiac amyloid deposition intense diffuse left ventricular myocardial uptake and quantitatively, a heart-to-contralateral lung (H/Cl) ratio of 1.76 (Figure 3).

Gene sequence analysis was positive for the pathogenic transthyretin (TTR) gene (p.Val142Ile), heterozygous, which is associated with autosomal dominant hereditary transthyretin-mediated amyloidosis (hATTR amyloidosis). Diagnostic test results are listed in Table 2.

**Table 2:** Diagnostic tests performed during hospitalization ATTR-CA, transthyretin cardiac amyloidosis

Test	Result
Electrocardiography (hospital day 1) (Figure 1)	Showed low-voltage QRS complexes. No discernible P waves. Ventricular escape rhythm with a ventricular rate of 34 bpm QRS duration of 120 milliseconds.
Chest X-ray (hospital day 1)	Showed cardiomegaly and bilateral pulmonary vascular congestion/pulmonary edema.
Transthoracic echocardiography (hospital day 2)	Severe left ventricular concentric hypertrophy (Figure 2, A) with an ejection fraction of 30%. Hypokinesis of the inferoseptal and basal inferolateral wall. Reduced tissue Doppler velocity with lateral E’ velocity of 5.71 cm/s and elevated E/lateral E’ of 17.3 (Figure 2, B). Reduce tissue Doppler velocity with medial E’ velocity of 4.54 cm/s and elevated E/medial E’ of 21.8 (Figure 2, C). Strain analysis showed a Global longitudinal score of -16% with apical sparing (Figure 2, D). Moderately dilated right ventricle, the systolic function was normal. Moderately dilated right and left atria Moderate thickening of the mitral valve with moderate regurgitation. Severe tricuspid regurgitation Trivial pericardial effusion.
99m-technetium-pyrophosphate scintigraphy (hospital day 8)	Showed myocardial greater than skeletal uptake with an increased heart-to-contralateral lung ratio of 1.76 (a cutoff value of >1.5 is strongly suggestive of ATTR-CA (Figure 3).
Electrocardiography (hospital day 10)	Normal sinus rhythm with a ventricular rate of 68 bpm Low voltage QRS complexes.
Genetic sequence analysis (hospital day 13)	A pathogenic variant, c.424G>A (p.Val142Ile), was identified in the transthyretin gene heterozygous mutation. The transthyretin gene is associated with autosomal dominant hereditary transthyretin-mediated amyloidosis.

While in the emergency room, the patient received an intravenous (IV) infusion of calcium gluconate, IV insulin with dextrose, oral potassium-binding resin for hyperkalemia, and furosemide 40 mg IV push for fluid overload. The patient was also started on a norepinephrine drip (starting at 5 mcg/kg/min) to maintain a MAP of >65 mmHg. An emergent transvenous pacemaker (TVP) was placed at a rate of 60 bpm for treatment of persistent profound bradycardia with a heart rate ranging between 30 and 40 bpm. Then, the patient was transferred to the cardiovascular intensive care unit for close monitoring and treatment.

The patient required multiple vasopressor support for refractory shock until day 7 and inotropic support from day 2 to day 3 to maintain goal MAP. The hospital course was also complicated by acute renal failure and hyperkalemia refractory to medical management requiring urgent hemodialysis and urinary tract infection, which was treated with ceftriaxone. Throughout hospitalization, she also received furosemide 80 mg IV push twice daily, which was gradually transitioned to furosemide 40 mg orally at the time of discharge. Eventually, the patient was hemodynamically stable. Follow-up labs showed improvement in renal function with a creatinine of 1.5 mg/dL and hyperkalemia resolved with potassium of 4.3 mmol/L. Post-treatment ECG was consistent with normal sinus rhythm. TVP was removed.

On hospital day 15, the patient was discharged on tafamidis 61 mg once daily, furosemide 40 mg once daily, aspirin 81 mg daily, apixaban 5 mg twice daily, and dapagliflozin 10 mg once daily with outpatient follow-up in the cardiology clinic. The patient was also enrolled in an amyloid program. Sacubitril/valsartan and AV-nodal blockers were discontinued and were advised to be avoided in the future. Prior to discharge, the patient was offered an automated implantable defibrillator (AICD) but she opted for a wearable cardioverter-defibrillator (WCD), which was arranged.

The patient presented for a follow-up two weeks after discharge and denied any symptoms or no shocks delivered by WCD. The result of the genetic study was discussed with the patient, including genetic counseling and screening of family members. The patient was scheduled for a closer follow-up and explained about aggressive disease progression.

## Discussion

**Figure 4:**
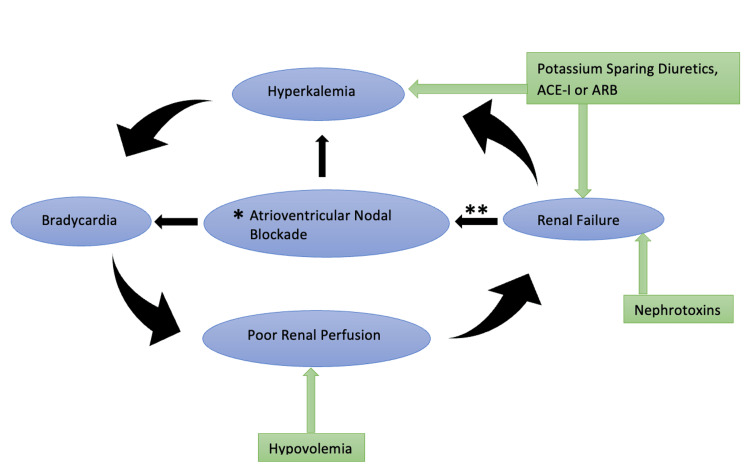
BRASH syndrome pathophysiology *Atrioventricular nodal-blocking agents such as beta-blockers, calcium-channel blockers, amiodarone, and digoxin **Serum level of renally excreted atrioventricular nodal blockers increases as the glomerular filtration rate decreases ACE-I, Angiotensin-converting enzyme inhibitors; ARB, Angiotensin-receptor blockers; BRASH, Bradycardia, Renal failure, Atrioventricular-nodal blockade, Shock, and Hyperkalemia. Image Credits: Nazima Khatun

The pathophysiology of BRASH syndrome is illustrated in Figure 4.

The clinical presentation of BRASH syndrome can be similar to AV-nodal blockade toxicity as well as isolated hyperkalemia. It is important to differentiate these diagnoses as definitive management will vary. The clinical history may provide important clues to differentiate them from each other. Beta-blocker toxicity is usually associated with hypoglycemia, while hyperglycemia is common with calcium-channel blocker toxicity. In isolated hyperkalemia, as the serum potassium level increases, progressive ECG changes occur with peak T wave then flattened P wave with PR prolongation followed by QRS widening and bradycardia. However, this progression is often absent in BRASH syndrome.

The severity of the presentation of BRASH syndrome ranges from asymptomatic bradycardia to multiorgan failure [3]. A high index of clinical suspicion for BRASH is required when encountering patients with refractory bradycardia and hyperkalemia [3]. The treatment approach is to break the vicious cycle by addressing several components simultaneously [1,3]. Profound bradycardia may fail to respond to standard therapy such as atropine, transcutaneous pacing, and medical management of hyperkalemia or AV-nodal blockade overdose [4]. Vasopressor support and early introduction of TVP may help improve the hemodynamic status of patients with BRASH [4]. 

In cardiac amyloidosis, ECG may show a low QRS voltage or pseudo-infarction pattern [6]. The common echocardiographic findings in cardiac amyloidosis are the presence of increased wall thickness and small chamber size of the left ventricle with systolic impairment, atrial enlargement, and restrictive pattern on Doppler, and the apical sparing pattern is a classic sign of ATTR-CA [6]. Cardiac magnetic resonance with late gadolinium enhancement is useful for diagnosing cardiac amyloidosis [6,7]. 99m-Technetium-pyrophosphate-scintigraphy is reported to have 100% specificity in diagnosing ATTR-CA [6]. The gold standard for diagnosing amyloidosis remains tissue staining. Congo red or Direct Fast Scarlet 4BS staining binds to deposit amyloid fibrils and, under polarized light microscopy, yields characteristic apple‐green birefringence. In addition, electron microscopy demonstrates randomly oriented and non‐branching fibrils [6]. Although tissue diagnosis remains the gold standard for diagnosis, positive 99m-Technetium-pyrophosphate-scintigraphy, in addition to the absence of detectable monoclonal protein, in urine or serum is diagnostic for ATTR-CA [6,7]. Genetic sequencing of the transthyretin gene helps identify variant-type ATTR-CA, identification of which should involve genetic counseling and potential screening of family members [7]. Early initiation of tafamidis on those with ATTR and NYHA class I to III symptoms appears to slow the disease progression [7]. 

Cardiac amyloid can cause atrial fibrillation/flutter, heart failure, and heart block if there are infiltrations in the conduction pathway. If the diagnosis of cardiac amyloidosis is not known in the patient, the treating physician may treat it like a case of heart failure. Implementing beta-blockers, angiotensin-converting enzyme inhibitors (ACE-I), and angiotensin-receptor blockers (ARBs) are standard care for heart failure, however, these medications can be harmful in cardiac amyloidosis and are keen to promoting BRASH syndrome, causing bradycardia, hypotension, and hyperkalemia. In our aging population, there is a high prevalence of kidney disease and polypharmacy. Therefore, it is not uncommon to have a patient with kidney disease on AV nodal blockade medications, as well as medications that can cause hyperkalemia. A thorough review of a patient’s medication list and their interactions is paramount. It is important to have BRASH on the differential if a patient presents with the symptoms and is on medications that can cause the syndrome.

## Conclusions

The BRASH syndrome can lead to life-threatening complications, including rapid progression to cardiac arrest, therefore, early recognition and aggressive management are crucial. We describe a case of ATTR-CA diagnosed when the patient presented with BRASH syndrome. ATTR-CA has been becoming common in the elderly and because ATTR amyloidosis predisposes them to BRASH syndrome, AV-nodal blockers should be cautiously prescribed to these patients.
